# Graphene Quantum Dots’ Surface Chemistry Modulates the Sensitivity of Glioblastoma Cells to Chemotherapeutics

**DOI:** 10.3390/ijms21176301

**Published:** 2020-08-31

**Authors:** Giordano Perini, Valentina Palmieri, Gabriele Ciasca, Marcello D’Ascenzo, Jacopo Gervasoni, Aniello Primiano, Monica Rinaldi, Daniela Fioretti, Chiara Prampolini, Federica Tiberio, Wanda Lattanzi, Ornella Parolini, Marco De Spirito, Massimiliano Papi

**Affiliations:** 1 Dipartimento di Neuroscienze, Università Cattolica del Sacro Cuore, 00168 Rome, Italy; giordano.perini@unicatt.it (G.P.); gabriele.ciasca@unicatt.it (G.C.); marcello.dascenzo@unicatt.it (M.D.); marco.despirito@unicatt.it (M.D.S.); 2 Fondazione Policlinico Universitario A. Gemelli IRCCS, 00168 Rome, Italy; jacopo.gervasoni@policlinicogemelli.it (J.G.); aniello.primiano@unicatt.it (A.P.); chiara.prampolini@unicatt.it (C.P.); federica.tiberio@unicatt.it (F.T.); wanda.lattanzi@unicatt.it (W.L.); ornella.parolini@unicatt.it (O.P.); 3 Institute for Complex Systems, National Research Council (ISC-CNR), Via dei Taurini 19, 00185 Rome, Italy; 4 Dipartimento di Scienze Biotecnologiche di Base, Cliniche Intensivologiche e Perioperatorie, Università Cattolica del Sacro Cuore, 00168 Rome, Italy; 5 Institute of Translational Pharmacology (ITF), Department of Biomedical Sciences, National Research Council (CNR), 00168 Rome, Italy; monica.rinaldi@ift.cnr.it (M.R.); daniela.fioretti@ift.cnr.it (D.F.); 6 Dipartimento di Scienze della Vita e Sanità Pubblica, Università Cattolica del Sacro Cuore, 00168 Rome, Italy

**Keywords:** nanomedicine, graphene quantum dots, glioblastoma, cancer treatment, synergistic therapy

## Abstract

Recent evidence has shown that graphene quantum dots (GQDs) are capable of crossing the blood–brain barrier, the barrier that reduces cancer therapy efficacy. Here, we tested three alternative GQDs’ surface chemistries on two neural lineages (glioblastoma cells and mouse cortical neurons). We showed that surface chemistry modulates GQDs’ biocompatibility. When used in combination with the chemotherapeutic drug doxorubicin, GDQs exerted a synergistic effect on tumor cells, but not on neurons. This appears to be mediated by the modification of membrane permeability induced by the surface of GQDs. Our findings highlight that GQDs can be adopted as a suitable delivery and therapeutic strategy for the treatment of glioblastoma, by both directly destabilizing the cell membrane and indirectly increasing the efficacy of chemotherapeutic drugs.

## 1. Introduction

Quantum dots (QDs) are semiconducting nanomaterials typically sized less than 20 nm in diameter [1]. In the last years, the interest in QDs has increased in different scientific branches due to their unique optical and electronic properties [2]. Particularly, graphene quantum dots (GQDs) have been recently studied in biomedical fields of applications, for their good biocompatibility and for their “molecule-like” features that characterize them over other QDs [3,4]. GQDs are fragments of graphene, a single-layer, two-dimensional carbon allotrope [5]. Graphene-based materials, especially graphene oxide (GO), have been widely employed in several biomedical applications as antibacterial agents [6,7,8], as scaffolds for bone regeneration [9,10], and as diagnostic tools [11,12]. Among graphene-based materials, GQDs are emerging as excellent candidates for bioimaging, in theranostic approaches, and, importantly, as drug-delivery agents [3].

More recently, GQDs have started to be exploited in the neuroscience field, specifically as nontoxic-labeling agents for human neural stem cells [13], without affecting cell self-renewal properties. Thanks to their extremely reduced size (<20 nm), GQDs can cross biological barriers [14], including the blood–brain barrier (BBB), which allows an extremely stringent and selective entry of the substances circulating in the blood toward the cerebral compartment. The capability of crossing the BBB is of particular relevance for the treatment of brain tumors, such as glioblastoma multiforme (GBM), the most aggressive subtype of glioma [15]. Indeed, the BBB prevents the delivery of chemotherapeutic drugs such as doxorubicin (Dox) [16] that can effectively kill GBM cells in vitro [17]. Nevertheless, Dox is not currently employed for its poor targeting in vivo, which could lead to an increased toxicity for healthy tissues. Recent works have also demonstrated how the surface chemistry of small particles plays a key role in the crossing of the BBB, in particular, when the surface is functionalized with carboxyl and amino groups [18]. 

Most studies focused on the efficacy of the conjugation of GQDs with Dox. Iannazzo [5] and colleagues synthesized a drug delivery system made of GQDs covalently bound with biotin. The receptor of biotin, as well as the folic acid receptor, is over-expressed on cancer cells. Dox was loaded on the system and its efficacy was evaluated on A549 cells. Cell viability was reduced by 35% due to the uptake of Dox inside cells. In another work, Wang and colleagues [19] conjugated GQDs with Dox and folic acid in order to obtain a nanoassembly capable of (1) targeted delivery to cancer cells, thanks to the increased expression of folic acid receptor, (2) real-time tracking of the assembly, thanks to GQDs, and (3) cytotoxic effect of Dox. The uptake of Dox was increased, thanks to the nanoassembly system. Nevertheless, cell viability measurements pointed out a reduction in the efficacy of the chemotherapeutic agent. Taken together, those pieces of evidence suggested that the conjugation of GQDs and Dox do not significantly increase the efficacy of the chemotherapeutic agent, despite increasing cellular specificity in the uptake of Dox. However, the conjugated nanocomplexes could not be stable and could reduce the effect of the chemotherapeutic agent itself [20]. For this reason, in our work we used GQDs as sensitizing agents for chemotherapeutic drugs, avoiding the formation of complexes.

In this work we employed three GQDs with different surface chemical functionalization: Green GQDs (Green-GQDs), with no surface-specific functionalization, carboxylated (COOH-GQDs), and aminated GQDs (NH_2_-GQDs). The biological effects of the three GQD compounds were tested and compared in two distinct neural cell lineages (U87 glioblastoma cells and mouse primary cortical neurons). We measured cell response after treatment with a combination of the three GQDs and Dox. The combined effect of GQDs and Dox was evaluated and it allowed observing a synergy of the two molecules on U87 cells in terms of effect on cell viability. Interestingly, the increased in vitro efficacy on glioblastoma cells correlated with the ability of GQDs to alter the cell membrane. The same synergistic effect was not observed on cortical neurons. Moreover, we demonstrated that the change in membrane fluidity is strictly related to the different surface charge of the three GQDs and, consequently, on cell permeabilization by different GQDs. The biophysical mechanism underlying the synergy of GQDs with the chemotherapeutic drug is a cornerstone for the development of new targeted delivery strategy aimed at crossing BBB.

## 2. Results

### 2.1. Characterization of GQDs 

**Figure 1:**
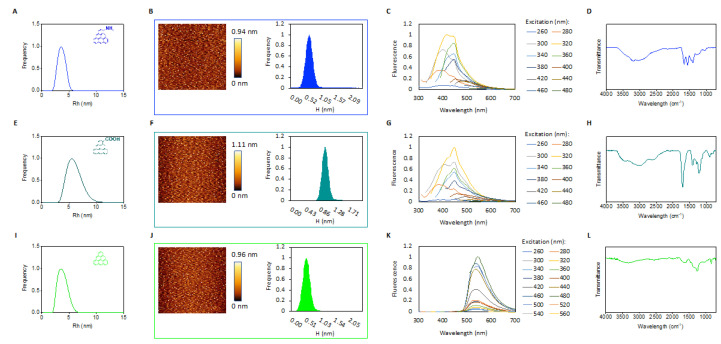
Characterization of the three functionalized graphene quantum dots (GQDs). Dynamic light scattering (DLS), atomic force microscopy (AFM imaging, height distribution, fluorescence spectra and Fourier transform infrared spectroscopy (FTIR) spectra of NH_2_-GQDs (**A**–**D**), COOH-GQDs (**E**–**H**) and Green-GQDs (**I**–**L**).

GQDs were characterized by dynamic light scattering (DLS), atomic force microscopy (AFM), fluorescence spectroscopy, and by attenuated total reflection-Fourier transform infrared spectroscopy (ATR-FTIR). DLS analysis was carried out on samples with a concentration of 100 µg/mL and showed that all particles have hydrodynamic radius below 10 nm (Figure 1A,E,I), thus outlining their comparable size. COOH-GQDs showed a broader intensity distribution (PdI) compared to the other particles, which is in accordance with what is reported on the technical datasheet. AFM imaging experiments were performed on samples diluted to a final concentration of 10 µg/mL (Figure 1B,F,J). AFM confirmed the size distribution obtained by DLS. Height distribution of each nanoparticle was measured, and the recorded height is comparable to data reported for graphene [7]. 

NH_2_-GQDs revealed an emission peak at 420 nm when excited at 320 nm (Figure 1C). COOH-GQDs showed an emission peak at 450 nm when excited at 320 nm (Figure 1G). NH_2_-GQDs and COOH-GQDs revealed similar emission peaks when excited at 320 nm (420 and 450 nm, respectively). Although these spectra look similar, amination resulted in an increased quantum yield for NH_2_-GQDs when compared to COOH-GQDs. Lastly, Green-GQDs showed an emission peak at 540 nm, in the region between green and yellow, when excited at 480 nm (Figure 1K). They also displayed a smaller peak at the same emission wavelength, if excited at 360 nm. For all the three particles, the full width at maximum height (FWMH) was approximatively 100 nm, as already reported for most of the carbon and graphene quantum dots [21]. Given the relationship between size and optical properties of GQDs [1], fluorescence intensity measurements resulted in accordance with DLS and AFM experiments [22].

The IR spectra of NH_2_-GQDs (Figure 1D) showed a band around ~3200 cm^−1^ that was associated with the N–H stretching vibration of amine groups [23]. The band at 1656 cm^−1^ corresponded to amide I (the –C=O stretch) and at 1536 cm^−1^ was assigned to amide II (the –C–N stretch and –C–N–H deformation) [24]. Finally, a band around 1403 cm^−1^ was due to C–N absorption. The IR spectra of COOH-GQDs (Figure 1H) showed characteristic IR frequencies of the carboxylic acid group, a broad band between 3000–2800 cm^−1^ for the O-H stretch, and a very strong band at 1705 cm^−1^ for the C=O stretch. The IR spectra of Green-GQDs (Figure 1L) showed a band at 3333 cm^−1^ due to O–H stretching vibrations, at 1613 cm^−1^ due to C=C bond and 1260 cm^−1^ epoxide C-O stretching vibrations. 

Taken together, spectroscopic and microscopic characterization techniques highlight how the three particles have comparable size distribution, but relevant differences in surface charge and FTIR spectra related to their surface chemistry. 

### 2.2. Cytotoxicity 

**Figure 2:**
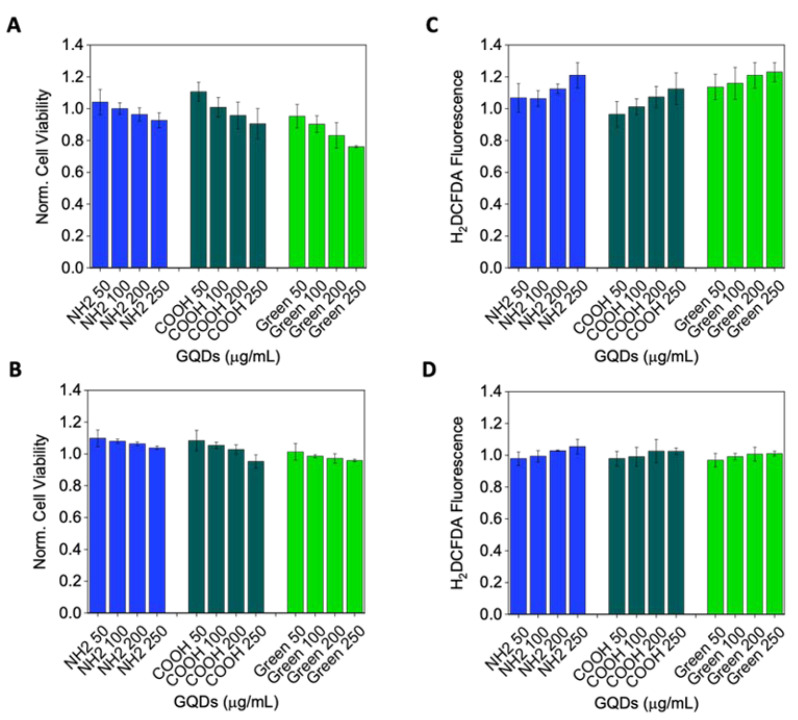
Toxicity of GQDs on U87 and cortical neurons after the treatment with GQDs. Cell viability normalized by control U87 untreated cells (**A**) and cortical neurons (**B**). Reactive oxygen species (ROS) production measured by fluorescence intensity of 2′,7′-dichlorodihydrofluorescein diacetate (H_2_DCFDA) for U87 (**C**) and cortical neurons (**D**) normalized by control cells.

We then investigated the cytotoxicity of GQDs on the U87 glioblastoma human cell line to study whether specific surface chemical functionalization could affect their biological effect. To compare results with a noncancerous cell system of the same anatomical compartment, cortical neurons were cultured and biocompatibility of GQDs was assessed by testing their effect on cell viability, using Celltiter Blue assay at different concentrations (250, 200, 100, and 50 µg/mL) after 24 h of treatment. Results are reported in Figure 2A,B for U87 and cortical neurons, respectively. Data are expressed as the mean values normalized to the untreated (control cells) ± standard deviation. A mild decrease in cell viability was found in U87 cells treated with Green-GQDs at the highest concentration (250 µg/mL), with a loss of viability around 20% (Figure 2A). However, Green-GQDs at 200, 100, and 50 µg/mL did not affect U87 growth. COOH-GQDs and NH_2_-GQDs did not decrease cell viability for U87 cells. In cortical neurons (Figure 2B) no significant reduction in cell viability was reported for all the GQDs at the four tested concentrations with respect to control cells, thus highlighting their high biocompatibility. 

Similar results have already been reported in previous works. Yuan and coworkers [25] successfully administered QDs with three different surface-functional groups to C6 glioma cells up to 200 µg/mL: Aminated, carboxylated, and dimethyl-formamide GQDs. In their study, no significant toxicity was pointed out. A mild reduction in cell viability was measured for all three GQDs at their highest concentration, in accordance with our results for Green-GQDs. Zhang and colleagues^13^ recently synthesized fluorescent GQDs in the absence of any coating or conjugation with macromolecules. When administered to human neural stem cells (hNSCs) to investigate uptake and biocompatibility, GQDs had no effect on cell viability even after 72-h exposure to a concentration of 200 μg/mL. The different response to the treatment with GQDs between cancer cells and physiological cells is still under investigation. While normal cells have mechanisms to protect themselves from toxic agents or growth-limiting conditions, cancer cells are more susceptible to the cytotoxic effects of drugs, in part due to the lack of checkpoints’ control mechanisms. Furthermore, important changes in the surface of malignant cells have been already observed, leading to a variety of properties that characterize their behavior and response [26].

In a previous work, Zhu and colleagues [21] synthesized green-photoluminescent GQDs by using a single-point solvothermal route, starting from graphene oxide, to use them for bioimaging of MG-63 human bone osteosarcoma cell line. In their work, cytotoxicity was investigated, and a lack of toxicity even at high concentrations of GQDs (up to 400 µg for 150 µL of medium) was pointed out. In the same study, Zhu confirmed biocompatibility of the GQDs synthesized on another cell line, MC3T3 (mouse osteoblast precursor), at the same concentrations. Wang and colleagues [19] tested cytotoxicity of carbon black-synthesized GQDs on different cell lines, namely HeLa cells, lung adenocarcinoma A549 cells, and human embryonic kidney cells HEK293A via 3-[4,5-dimethylthiazole-2-yl]-2,5-diphenyltetrazolium bromide (MTT) test. In that study, GQDs did not induce considerable cytotoxicity to all three cell types. However, they tested a concentration of 20 μg/mL, which was too low when compared to our range. For the toxicity of surface-functionalized GQDs, Wang and coworkers [27] synthesized nitrogen-doped carbon dots via one-pot hydrothermal treatment of folic acid aqueous phase synthesis and evaluated the effect on U87 glioblastoma cells. Cell viability was not affected in the range of 10 μg/mL to 1000 μg/mL, and no obvious morphological changes were observed after the treatment of cells with GQDs. This is in accordance with the high biocompatibility we observed for aminated GQDs, even at high concentration (250 μg/mL). Carboxylated GQDs were previously synthesized by Nurunnabi and colleagues [28] by exfoliation of carbon fibers in acidic media, and cell viability was measured by MTT on human cervix KB HeLa-derived cells, epithelial human breast cancer MDA-MB-231, A549 cancer cells, and Madin-Darby Canine Kidney (MDCK) normal cells. GQDs showed the best biocompatibility on MDCK cells among the lines, while the most evident cytotoxicity, around 40% at the highest concentration (500 μg/mL), was in KB cells. Their evidence strongly support our data, since at 250 μg/mL there was no significant toxicity reported for carboxylated GQDs on the four different cell lines. 

One of the best-described intracellular stress mechanisms exerted by nanoparticles on cells is the induction of reactive oxygen species (ROS) production. This property is believed to lie in the surface characteristics and in the composition of the nanomaterials [29]. Previous studies conducted on graphene showed a dose-dependent production of ROS on eukaryotic cells, which was related to the surface defects of the material [30]. Moreover, Gurunathan and colleagues [31] investigated the antibacterial activity of GO and reduced GO on *Pseudomonas aeruginosa*. The levels of ROS in GO- and reduced GO-treated cells were significantly higher when compared to the level of ROS in control cells throughout their experiment. For these reasons, even if no significant toxicity was present on both cell lines, we further investigated the formation of ROS, in order to reinforce the previous promising cell viability data. For this purpose, we employed 2′,7′-dichlorodihydrofluorescein diacetate (H_2_DCFDA) on both cell lines after 24 h of treatment with the four previous concentrations of the three GQDs. No significant changes in the fluorescence intensity of H_2_DCFDA were observed for both U87 glioblastoma cells (Figure 2C) and cortical neurons (Figure 2D). Wu and colleagues [32] previously compared the production of ROS on breast cancer MCF-7 cells treated with GO and GQDs. They showed that while GO induced significant production of ROS, GQDs did not affect cells, remaining similar to control untreated cells. Markovic [33] demonstrated that the production of ROS induced by GQDs was related to their photoexcitation at 470 nm, while no alteration in reactive species was present without the irradiation. 

It has also been reported that various terminal end groups on functionalized QDs can result in a variable level of inflammatory responses [34]. Inflammatory potential of the three different GQDs was assessed by measuring the release of the pro-inflammatory cytokines IL-6 and TNF-α and the anti-inflammatory cytokine IL-10. As a primary mediator of the inflammatory response, IL-6 is very important in the pathogenesis of many types of cancer. IL-6 is abundantly produced by glioma cells and upregulation of IL-6 in gliomas is associated with the grade of malignancy and could play an important role in gliomagenesis, angiogenesis, and metastasis [35]. U87 cells express high amounts of IL-6, as do highly aggressive glioblastomas [36,37]. A correlation between IL-6 gene expression and shortened survival in glioblastoma patients has been demonstrated [38]. Pro-inflammatory cytokines such as the TNF-α have been reported as stimulators of apoptotic mechanisms [39] and clinical evidence supports the concept that an excess of TNF-α plays a central role in neuronal degeneration [40].

**Figure 3:**
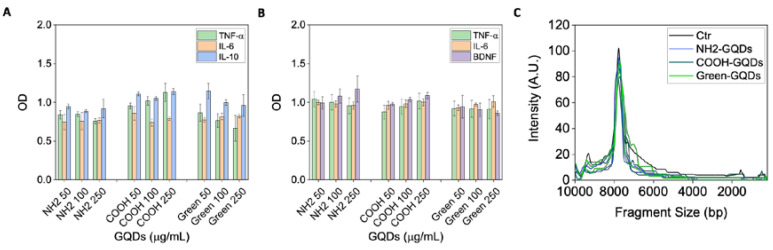
Effect of GQDs on secretion of cytokines, as reported in the relative legend, for U87 (**A**) and cortical neurons (**B**). Data are expressed as the mean normalized to the untreated control cells ± standard deviation. Line profile of DNA fragmentation for U87 and cortical neurons (**C**).

We then investigated the effect of GQDs on the release of these cytokines in U87 human malignant glioma exposed to the GQDs for 24 h over a broad concentration range (250, 100, and 50 µg/mL). ELISA assays demonstrated that no increase in the secretion of the pro-inflammatory cytokines TNF-α and IL-6 could be observed for any GQDs type at any concentration compared with the level produced by untreated control cells (Figure 3A). Rather, a mild decrease in IL-6 levels was found in U87 cells treated with GQDs, although the difference was not statistically significant. No reduction in the release of the anti-inflammatory cytokine IL-10 was observed overall, indicative of the good compatibility of the modified GQDs in terms of inflammatory properties (Figure 3A). The good biocompatibility of the three modified GQDs was not cell-specific, which was evidenced by the similar results gained from the primary cortical-derived neurons. Compared with untreated cells, there were no differences in either the levels of IL-6 or TNF-α in GQDs-treated cortical neurons (Figure 3B). These data indicate that GQDs’ exposure in cortical neurons had no effect on secretion of TNF-α and IL-6 cytokines that are central to neuroinflammatory processes [41]. Brain-derived neurotrophic factor (BDNF) is a specific marker of chemical neurotoxicity. It has been demonstrated that the release of this protective neurotrophin into the culture medium occurs in response to sub-cytotoxic levels of known neurotoxic chemicals [42]. Thus, the release of BDNF protein from cortical neurons was also tested by ELISA assay. The analysis of BDNF secretion is presented in Figure 3B and shows that the level of this neuroprotective—neurotrophic factor did not increase in response to GQDs’ exposure, confirming their good profile of biocompatibility.

Furthermore, DNA integrity was unaffected by any of the tested GQD treatments, as detected by the DNA migration patterns on agarose gel (Figure 3C).

### 2.3. Synergy of GQDs and Dox 

**Figure 4:**
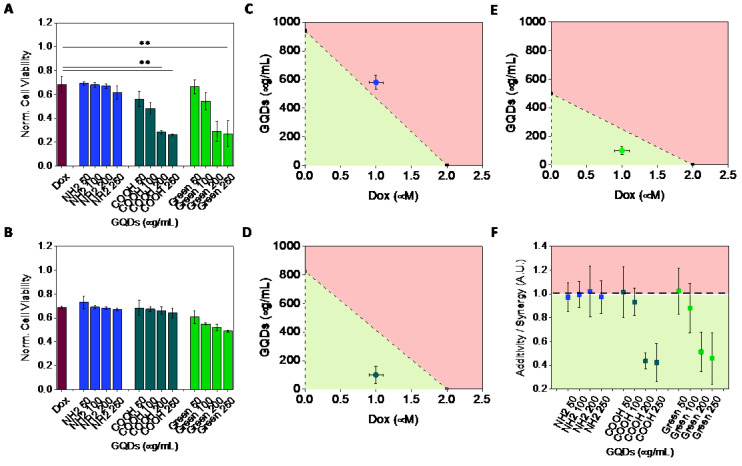
Synergy of GQDs and doxorubicin (Dox). Normalized cell viability for U87 (**A**) and cortical primary neurons (**B**) after the treatment with GQDs and Dox separately. Isoboles’ graphs of Dox with NH_2_, COOH, and Green-GQDs, respectively, on U87, to determine the presence of synergistic effects (**C**–**E**). Synergy expressed as the ratio between the theoretical additive effect of GQDs with Dox and the measured effect (**F**). ** *p* < 0.01, one-way ANOVA, and Tukey post hoc test.

Given the lack of toxicity of GQDs alone, we investigated if they could be used as sensitizing agents for doxorubicin (Dox)-based treatments. First, we found the concentration of Dox inhibiting 50% of cell growth (IC50), which resulted to be 2 µM (data not shown). For further experiments, we set our working concentration of Dox at half of IC50. The chemotherapeutic agent alone exhibited a toxicity of around 40% on both cortical neurons (Figure 4A) and U87 (Figure 4B) at half IC50. We then tested the combined effect of GQDs and Dox by administering them to cells separately. We carried out this experiment in two different conditions. In the first, after the treatment with GQDs, Dox was added without washing away the nanoparticles. In the second, after the treatment with GQDs, they were washed away to avoid direct interactions between QDs and the drug and then administered doxorubicin. No differences were observed between the two different protocols. Therefore, by washing GQDs before Dox administration, we could exclude the hypotheses of GQDs’ and Dox’s extracellular interaction [43]. When pretreated with NH_2_-GQDs, no changes in cell viability were measured for all the cell lines investigated (Figure 4A,B). The combination of COOH-GQDs or Green-GQDs at 200 and 250 μg/mL with Dox caused a reduction in cell viability of U87 from 60% to 20% (Figure 4A). On the other hand, cortical neurons were not affected by Dox after pretreatment with COOH-GQDs or Green-GQDs (Figure 4B).

We then verified the synergy of the two molecules by creating isoboles’ graphs (Figure 4C). In these graphs the concentration of GQDs and Dox capable of reducing cell viability by 50% is connected by a dashed line. This line defines two areas area in which there is an agonist effect (below the line) or an additive effect (above the line). The treatment with NH_2_-GQDs and Dox was not synergistic for U87 cells, as highlighted in the isoboles (Figure 4C). COOH-GQDs (Figure 4D) and Green-GQDs (Figure 4E) with Dox on U87 cells showed a synergistic effect. We then calculated the ratio between the theoretical additive effect of GQDs with Dox and the measured effect (Figure 4F), highlighting the synergy of COOH-GQDs and Green-GQDs at 200 and 250 μg/mL with Dox.

**Figure 5:**
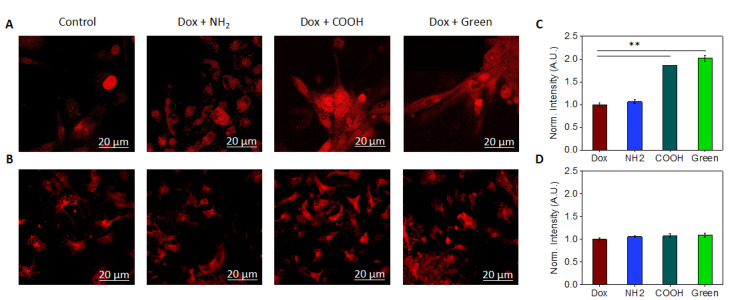
Dox uptake inside U87 (**A**) and cortical primary neurons (**B**) after the pretreatment with GQDs at 250 µg/mL. Fluorescence intensity of Dox inside U87 cells (**C**) and inside cortical neurons (**D**). ** *p* < 0.01, one-way ANOVA and Tukey post hoc test.

We investigated whether the synergistic effect was related to an increase in the uptake of Dox inside U87 cells. Confocal microscopy was carried out on U87 cells and cortical neurons and results confirmed, consistent with our model, the increase in the uptake of Dox for U87 cells treated with COOH and Green-GQDs (Figure 5A,C). As expected, no differences were observed in the fluorescence intensity of Dox for cortical neurons with or without the treatment with GQDs (Figure 5B,D). The enhanced effect of chemotherapeutic drugs with GQDs has recently been described also by other groups. Sui and coworkers [20] pointed out the increased efficacy of cisplatin on different cell lines when treated with GQDs: Breast cancer MCF-7 cells, A549 cells, HeLa cells, and human gastric cancer MGC-803 cell line. In this work, the combination of cisplatin and GQDs led to more cells arrested at gap2/mitotic (G2/M) phase with respect to untreated cells, together with an increase of apoptotic bodies. However, the reduction in cell viability was mild, even though the uptake of cisplatin was found to be increased.

### 2.4. Analysis of the Interaction Mechanism between GQDs and Cell Compartments

As suggested by Sui and coworkers [20], the combined effect of GQDs and the chemotherapeutic agent could be due to an extracellular interaction between the two molecules. After the interaction, the nanocomplex could easily enter cells and release the drug, thus increasing its efficacy compared to the drug alone. However, this mechanism could not be stable and could reduce the effect of the chemotherapeutic agent itself. Therefore, to exclude the hypothesis of a synergistic effect mediated by an extracellular interaction between the two molecules, we measured cell viability of U87 in two different conditions. In the first condition, GQDs and Dox were co-administered to glioblastoma cells, in order to allow an interaction between the two molecules. In the second, GQDs were washed away and Dox was administered separately to avoid extracellular interactions between the two molecules. Cell viability was measured in both conditions, and no differences were observed (data not shown), thus excluding the hypothesis of a synergistic effect mediated by an extracellular interaction between the particles.

Another hypothesis could include an interaction between GQDs and cell membrane that could change membrane permeability, increasing the entrance of the chemotherapeutic agent inside cells [20]. Therefore, we evaluated the alterations of membrane permeability of U87 and cortical neurons after the treatment with GQDs [20]. For this purpose we labeled cells with Laurdan [44] that can be used to describe the lipid-phase state in membranes. Membrane fluidity can be measured by several techniques, such as nuclear magnetic resonance [45], diffusion of membrane molecules [46], and by fluorescence due to polarity-sensitive probes (Laurdan) [44]. When compared to other methods, polarity-dependent probes have a high sensitivity for differences in the packing of lipid bilayers in gel/liquid phases. Furthermore, among different fluorescent probes, such as the aminonaphthylethenylpyridinium dye di-4-ANEPPDHQ, Laurdan has shown its higher sensitivity [47].

**Figure 6:**
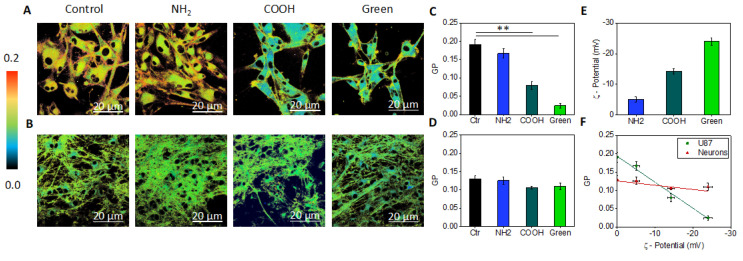
Membrane fluidity measured through generalized polarization (GP) of Laurdan emission for U87 (**A**) and cortical neurons (**B**). GP values measured for U87 (**C**) and cortical neurons (**D**). The ζ-potential of GQDs (**E**). Correlation between ζ-potential and GP for U87 (in green) and cortical neurons (in red) (**F**). ** *p* < 0.01, one-way ANOVA and Tukey post hoc test.

Membrane fluidity quantified by Laurdan is given by calculation of the generalized polarization (GP) index, which goes from −1 (highest membrane fluidity) to +1 (lowest membrane fluidity) [48]. Confocal microscopy measurements of Laurdan fluorescence pointed out alterations in membrane fluidity on U87 cells, but not in cortical neurons (Figure 6A,B). In the presence of COOH and Green-GQDs, a significant reduction in GP was measured, indicating an increase in membrane fluidity compared to untreated cells. When treated with 250 μg/mL of NH_2_-GQDs, U87 did not change membrane fluidity (Figure 6C). This could be due to positively charged groups [49], such as amino groups, which exhibit a stronger affinity for the negatively charged cell membrane, accounting for a higher cellular uptake [20]. Membrane fluidity of cortical neurons remained the same after the treatment with all the GQDs at their highest concentration (250 μg/mL, Figure 6D).

Previous experimental [50] and molecular simulation [51] studies demonstrated the capability of graphene-based materials to interact with cell membrane. Tu and colleagues [52] performed molecular dynamics’ simulations on graphene sheets and *E. coli* membranes. In their work, they observed three different modes of interaction between the two components: The swing mode, the insertion, and the extraction. In particular, in the insertion mode, at first, van der Waals attractions between lipid heads and the edge of graphene trap the latter. This evidence strongly indicates that the surface net charge of graphene edges influences its intercalation between lipid bilayer. Then, hydrophobic interactions between the inner core of graphene and the lipid tails take over. After a few nanoseconds, graphene nanosheets are inserted between the membrane. A similar molecular dynamics’ approach was carried out by Titov [53]. In its model, rectangular graphene sheets of 5.9 × 6.0 nm^2^ were incorporated between the lipid bilayer of cell membrane like a sandwich. Differently from Tu, Titov and colleagues started their simulation by putting micelles near the nanosheets. As expected, in this case, hydrophobic interactions took over van der Waals attractions from the beginning, driving graphene inside the bilayer. By steered-molecular dynamics, they were able to calculate the binding energy between the two components, which resulted around 42 kcal/mol. Sun and colleagues experimentally tested the interaction between nanosheets of GO and cell membrane [50], confirming their interaction. Interestingly, in a study by Li, Yuan, and von dem Bussche [51], both computational and experimental tests were performed. Coarse-grained and all-atom molecular dynamics’ studies revealed specific parameters involving the intercalation of graphene inside cell membrane. At first, edge asperities of graphene interact with membranes, in a mechanism called piercing. In their work, Li and colleagues observed that the rate of energy change during piercing can be expressed as a function of the penetration depth of graphene. After piercing of membranes, the rest of graphene is energetically driven between the bilayer, further confirming the model of Tu and Titov. This evidence strongly indicates that, at first, the surface net charge of graphene edges influences its intercalation between lipid bilayer.

Furthermore, glioblastoma multiforme shows an upregulation of low-density lipoprotein receptor (LDLR), unlike neurons [54]. Zhang and Monteiro-Riviere demonstrated the capability of carboxylated, negatively charged QDs to easily interact with cell membrane via LDLR-mediated endocytosis [55]. These results are in accordance with our experimental evidence and strongly indicate the different GQDs–membrane interaction in the two neural lineages.

**Scheme 1:**
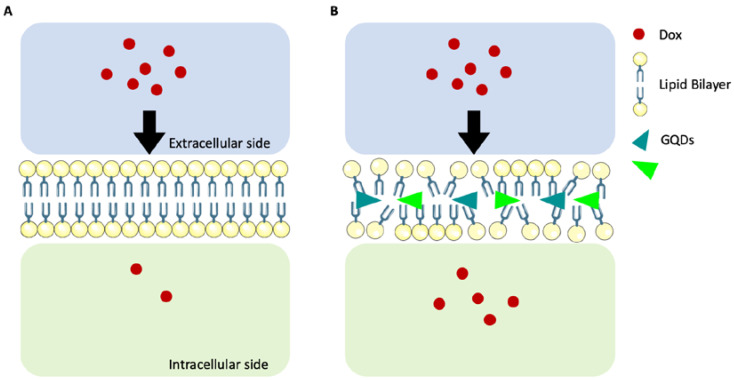
Synergistic mechanism between GQDs and Dox on U87. When Dox approaches cells, its uptake increases with the increase in membrane permeability. The amount of Dox uptake inside an untreated cell (**A**) is less than that of cells pretreated with COOH and Green-GQDs (**B**).

Therefore, we expected a correlation between the surface net charge of GQDs and alterations in membrane fluidity. Zeta potential measurements were carried out on the three GQDs (Figure 6E). Green-GQDs displayed a surface charge of −22.7 ± 1.14 mV, COOH-GQDs of −14.23 ± 0.81 mV, and NH_2_-GQDs of −5.08 ± 0.86 mV. This large change of the zeta potential for the surface-functionalized GQDs is due to the presence of the amino and carboxyl groups [56]. Likewise, the highly negative zeta potential of Green-GQDs was, as expected, in accordance with what was previously reported for GO, since both particles present a similar surface chemical composition [57]. The diverse surface net charges were related to the reduction of the GP for U87 cells, but not for cortical neurons (Scheme 1).

Taken together, those findings strongly indicate that different surface charges drove van der Waals interactions between GQDs and U87 cell membrane. Those interactions alter cell membrane permeability, as demonstrated in this work for the first time, facilitating the entrance of Dox (Figure 5), thus unfolding the synergistic effect between GQDs and the antitumor molecule.

## 3. Discussion 

The results obtained in this study demonstrate that the diverse functionalization of the surface chemistry of the GQDs significantly change the biological effects they exert on neural cells. All the tested GDQ species displayed high biocompatibility and low cytotoxicity, as they did not evoke ROS production, neuroinflammatory molecules’ secretion’ or DNA fragmentation in vitro. A minor effect on cell viability was induced exclusively by the Green-GQD at the highest concentration on U87 glioblastoma cells.

When Dox was administered to cells after 24 h of treatment with GQDs, no significant changes in cell viability of cortical neurons were measured when compared to the effect of Dox alone. Differently, U87 cells pretreated with Green-GQDs and COOH-GQDs at 200 and 250 µg/mL showed a significant reduction in cell viability when compared to Dox alone, indicating a synergistic effect between the two GQDs and Dox. The reduction in cell viability was related to an increase in the uptake of Dox. The pretreatment with NH_2_-GQDs did not affect cell viability and Dox uptake for both cortical neurons and U87 cells exposed to Dox. To understand the mechanism underlying the synergy between GQDs and Dox, we investigated, for the first time, changes in membrane permeability after the treatment with GQDs. U87 cells showed a significant increase in membrane permeability when Green-GQDs and COOH-GQDs were administered. As expected, NH_2_-GQDs did not affect membrane permeability. Neurons’ fluidity remained similar to that of control cells for all three GQDs. The changes in membrane fluidity for U87 cells were correlated to the surface net charges of the GQDs, in accordance with literature studies reporting a dependence on van der Waals interactions between graphene and cell membranes. Taken together, these results indicate a highly cell-specific and chemistry-dependent mechanism of action for GQDs, thus suggesting strong potential applications for Green and COOH-GQDs in drug-delivery systems for their capability of changing membrane permeability. On the other hand, since NH_2_-GQDs did not affect cell membrane or cell viability, they could be used for future bioimaging applications.

## 4. Materials and Methods

### 4.1. Characterization of GQDs

COOH-GQDs and NH_2_-GQDs GQDs in aqueous solution with a concentration of 1 mg/mL were purchased from ACS Material. Green-GQDs were purchased in powder (50 mg) from Sigma-Aldrich and dissolved in ultrapure water at a final concentration of 1 mg/mL. Fluorescence intensity spectra were obtained by using a Cytation 3 Cell Imaging Multi-Mode Reader (Biotek, Terrebonne, QC, Canada) [10] using excitation wavelengths from 260 to 600 nm with a step of 20 nm and reading the emission from 300 to 700 nm with a step of 5 nm. Fluorescence intensity spectra were normalized to their corresponding maximum emission. The 100 μL of samples with a concentration of 10 µg/mL were deposited on sterile mica slides and air-dried overnight for atomic force microscopy imaging (AFM) with a NanoWizard II (JPK Instruments AG, Berlin, Germany) [58]. The images were acquired using silicon cantilevers with high aspect-ratio conical silicon tips (CSC36 Mikro-Masch, Tallinn, Estonia) characterized by an end radius of about 10 nm, a half conical angle of 20°, and a spring constant of 0.6 N/m. Small scan areas (3 × 3 µm) were imaged. Dynamic light scattering and ζ-potential were performed with Zetasizer Nano ZS (Malvern, Herrenberg, Germany), equipped with a 633-nm He−Ne laser and operating at an angle of 173° [59]. UV-transparent cuvettes (Malvern, Herrenberg, Germany) were used for experiments with a sample volume of 500 µL and a concentration of 100 µg/mL. The measurements were performed at a fixed position (4.65 mm) with an automatic attenuator. For each sample, three measurements were averaged, the diffusion coefficient D was retrieved through cumulants’ analysis from autocorrelation functions. The equivalent hydrodynamic radius (Z-average size) was obtained by the Stokes−Einstein equation. Data analysis was performed by Malvern Zetasizer software. The chemical analysis of the GQDs was carried out using attenuated total reflectance-Fourier transform infrared spectroscopy (ATR-FTIR) by Spectrum One spectrometer (Perkin Elmer, Waltham, MA, USA). The material under investigation was directly laid upon the ATR crystal and the spectra were recorded in the wave number range of 4000–550 cm^−1^.

### 4.2. Cell Cultures

U87 human glioblastoma cells were purchased from the American Type Culture Collection, (ATTC). Cells were maintained in Dulbecco’s modified Eagle’s medium (Sigma-Aldrich, St. Louis, MO, USA) supplemented with 10% fetal bovine serum (FBS, EuroClone, Milan, Italy), 2% penicillin-streptomycin (Sigma-Aldrich), and 2% L-glutamine (Sigma-Aldrich).

Primary cultures of cortical neurons were obtained from E15-18 C57BL/6 mice embryos as described previously [60] and in accordance with the Ethics Committee of the Catholic University and complied with Italian Ministry of Health guidelines, with national laws (Legislative Decree 116/1992), and European Union guidelines on animal research (No. 86/609/EEC). Briefly, the mouse cortex was dissected in cold CMF-HBSS (Ca^2+^ and Mg^2+^ free Hank’s balanced salt solution containing 1 mM pyruvate, 15 mM 4-(2-hydroxyethyl)-1-piperazineethanesulfonic acid, HEPES, 10 mM NaHCO_3_). Tissues were then incubated for 10 minutes at 37 °C in phosphate buffered saline (PBS) containing trypsin-ethylenediaminetetraacetic acid (0.025%/0.01% wt/vol; Biochrom AG, Berlin, Germany), and the tissue was mechanically dissociated at room temperature with a fire-polished Pasteur pipette. The cell suspension was harvested and centrifuged at 235 *g* for 8 min. The pellet was suspended in 88.8% Minimum Essential Medium (Biochrom), 5% fetal bovine serum, 5% horse serum, 1% glutamine (2 mM), 1% penicillin-streptomycin-neomycin antibiotic mixture (Invitrogen, Carlsbad, CA, USA), and glucose (25 mM). Cells were plated at a density of 10^5^ cells/mL on a 24-well plate precoated with poly-L-lysine (0.1 mg/mL; Sigma, St. Louis, MO, USA). Twenty-four hours later, the culture medium was replaced with a mixture of 96.5% neurobasal medium (Invitrogen), 2% B-27 (Invitrogen), 0.5% glutamine (2 mM), and 1% penicillin-streptomycin-neomycin antibiotic mixture. After 72 h, this medium was replaced with a glutamine-free version of the same medium, and the cells were grown for 10 more days before experiments. All cell lines were cultivated in T75 flasks and kept at 37 °C in 5% CO_2_ humidity.

### 4.3. Cell Viability

For cell viability measurements, U87 human glioblastoma cells and cortical primary neurons were seeded on 96-well plates at the concentration of 1 × 10^5^/mL. Cells were treated, alternatively, with the three different GQDs (Green-GQD, COOH-GQDs and NH_2_-GQDs), each at four different concentrations: 250 μg/mL, 200 μg/mL, 100 μg/mL, and 50 μg/mL for 24 h. After the treatment, the medium containing GQDs was carefully washed and replaced with 120 μL of fresh medium containing 20 μL of CellTiter-Blue (Promega, Madison, WI, USA) and incubated in the dark at 37 °C in 5% CO_2_ for 2 h. Fluorescence intensity was recorded with Cytation 3 Cell Imaging Multi-Mode Reader by exciting at 550 nm and reading the emission at 600 nm.

To assess the synergy between GQDs and Dox, cells were seeded on a 96-well plate, as described above, for 24 h. Cells were then treated with the three different GQDs at four different concentrations: 250 μg/mL, 200 μg/mL, 100 μg/mL, and 50 μg/mL for 24 h. After the treatment, the medium containing GQDs was carefully washed and replaced with 120 μL of fresh medium containing 1 μM Dox, and cells were grown for a further 24 h. After the treatment, the medium containing GQDs was carefully washed and replaced with 120 μL of fresh medium containing 20 μL of CellTiter-Blue (Promega) and fluorescence intensity was recorded as previously described. In both cases, results were compared with control (untreated) cells.

### 4.4. ROS Detection 

For the detection of ROS, the fluorinated derivative of 2′,7′-di-chlorofluorescein (H_2_DCFDA) was employed. This probe is nonfluorescent until the acetate groups are removed by intracellular esterases and oxidation occurs within cells. Thus, oxidation can be detected by monitoring the increase in fluorescence intensity. Cells were seeded on 96-well plates at the concentration of 1 × 10^5^ cells/mL. After 24 h of incubation at 37 °C, 5% CO_2_, cells were treated with the three different GQDs at four different concentrations: 250 μg/mL, 200 μg/mL, 100 μg/mL, and 50 μg/mL for 24 h. After the treatment, the medium containing GQDs was carefully washed and replaced with PBS containing 10 μM H_2_DCFDA. Cells were incubated for an additional hour at 37 °C in 5% CO_2_. PBS containing H_2_DCFDA was then removed and cells were resuspended in complete medium. Fluorescence intensity of H_2_DCFDA was recorded by using a Cytation 3 Cell Imaging Multi-Mode Reader by exciting at 495 nm and recording emission at 528 nm. 

### 4.5. ELISA Assay

U87 human glioblastoma cells and cortical neurons were seeded on a 24-well plate at the concentration of 0.1 × 10^6^ cells/mL. Cells were treated with the three different GQDs at different concentrations: 250 μg/mL, 100 μg/mL, and 50 μg/mL for 24 h. After the treatment, the medium from treated and untreated cell cultures was collected, centrifuged at 1200 rpm for 5 min. and stored at −80 °C until use. Tumor necrosis factor-α (TNF-α), interleukin-6 (IL-6), interleukin-10 (IL-10), and brain-derived neurotrophic factor (BDNF) releases were measured in culture medium using human or mouse ELISA development kits (PeproTech^®^ EC Ltd., UK for TNF-α, IL-6 and IL-10; MyBioSource, Inc., San Diego, CA, USA for BDNF) according to the manufacturer’s instructions. Cell supernatants and recombinant standards were serially diluted in 1 × PBS/0.05% Tween-20/0.1% bovine serum albumin (BSA) (Sigma-Aldrich^®^, Dorset, UK) and added to the microplates. Factors’ binding was detected by biotin-avidin detection step, followed by chromogen 2,2′-azino-bis (3-ethylbenzothiazoline-6-sulphonic acid (Sigma-Aldrich^®^, Dorset, UK) incubation. Color development was monitored at 405 nm.

### 4.6. Statistical Analysis

All measurements were performed in triplicate, and data are reported as mean ± standard deviation. Statistical analysis was performed using one-way ANOVA, followed by Tukey’s post hoc test. Differences were considered significant when *p* < 0.01 (**). 

### 4.7. DNA Fragmentation

Cells were seeded on 6-well plates using a seeding density of 0.1 × 10^6^ cells/mL for U87 cells and cortical neurons. Cells were then incubated at 37 °C in a humidified hood with 5% CO2 for 24 h, then treated, alternatively, with the three different GQDs (namely, green GQD, COOH-GQDs, and NH2-GQDs) for 24 h. In particular, U87 human glioblastoma cells were treated with 250 μg/mL and 100 μg/mL of either of the three GQDs’ species, while cortical neurons were treated with 250 μg/mL and 50 μg/mL of either of the three GQDs’ species. Cells receiving a mock treatment of medium without GQDs served as controls in all experiments. DNA was extracted from all cells using standard protocols. Briefly, cells were lysed with 1 mL of SE buffer (NaCl 75 nM, Na_2_ ethylenediaminetetraacetic acid, EDTA, 25 nM, pH 8) supplemented with 2% sodium dodecyl sulfate (SDS) and 200 µg of proteinase K, for 30 min at 65 °C. After protein salting out with a saturated NaCl solution, DNA was isolated adding 2.5 volumes of absolute ethanol, washed twice in 70% ethanol, dried, and eluted in deionized bi-distilled water. DNA was quantified using a UV spectrophotometer (Beckman Coulter DU 800). Then, 250 ng of each sample were loaded on a 1.5% agarose gel to assess the integrity through electrophoresis.

### 4.8. Confocal Microscopy

To perform confocal microscope analysis, U87 and cortical neurons were plated on sterile chamber slides (Ibidi) at a concentration of 1 × 10^6^ cell per mL and then incubated at 37 °C. After 24 h of incubation, GQDs were administered to cells at the concentration of 250 μg/mL for a further 24 h. For both 6-dodecanoyl-2-dimethylamino-naphthalene (Laurdan) measurements and Dox uptake (Sigma-Aldrich), GQDs were carefully washed away with PBS, and cells were resuspended in fresh medium containing Laurdan or Dox at the following final concentrations. For Laurdan measurements, a stock solution of Laurdan 1 mM in DMSO was diluted 1:1000 in DMEM and administered to cells. For Dox uptake measurements, Dox was diluted from a stock solution 1.5 mM to a final concentration of 1 µM in DMEM and administered to cells. 

Confocal microscopy measurements of Laurdan and Dox uptake were carried out using an inverted microscope (Nikon A1 MP+, Nikon, Japan) equipped with a 60× oil immersion objective [7]. Images were acquired at 37 °C for all measurements. For Laurdan excitation, cells were imaged with a wavelength of 400 nm. Laurdan is a fluorescent probe with a hydrophilic head and a hydrophobic tail. Thanks to its dipole moment between the head and the tail, Laurdan is sensitive to the polarity of the environment, thus displaying a red shift of the emission in polar solvents, with respect to non-polar solvents [61]. This phenomenon is generally defined as solvent relaxation [44]. Thus, Laurdan is capable of detecting and distinguishing gel phase and liquid phase of cell membrane.

Laurdan intensity images were recorded simultaneously with emissions in the ranges of 425–475 nm (gel phase) and 500–550 nm (liquid phase). To quantify the images collected in this way, Fiji (ImageJ) software was used [62]. The intensities of the two different channels were calculated, and the generalized polarization (GP) index was calculated as follows [48]:

(1) $$\mathrm{GP}=\frac{I_{G}-I_{R}}{I_{G}+I_{R}}$$

where I_G_ and I_R_ are the intensities of the gel-phase and liquid-phase emission channels, respectively.

For Dox uptake measurements, images were acquired after 1 h of incubation with Dox [63]. The excitation and emission wavelengths were, respectively, 488 nm and 590 nm. To quantify Dox uptake, Fiji (ImageJ) software was used [62] by measuring the fluorescence intensities of Dox 1 µM in cells [19]. Fluorescence intensity of control (untreated) cells was subtracted in order to exclude autofluorescence, so that the measured fluorescence was due to doxorubicin. Data were normalized by cells treated with chemotherapeutic agent alone.
